# 5-FU induced cardiotoxicity: case series and review of the literature

**DOI:** 10.1186/s40959-019-0048-3

**Published:** 2019-09-06

**Authors:** Cai Yuan, Hiral Parekh, Carmen Allegra, Thomas J. George, Jason S. Starr

**Affiliations:** 1 Division of Hematology & Oncology, Department of Medicine, University of Florida, 8962 SW 73rd Lane, Gainesville, FL 32608 USA; 2 Division of Hematology & Oncology, Mayo Clinic, Jacksonville, FL USA

**Keywords:** Fluoropyrimidine, 5-FU, 5-fluorouracil, Cardiotoxicity, Cancer, Colorectal cancer, Antimetabolite, Toxicity, Cancer complications

## Abstract

**Background:**

5-Fluorouracil (5-FU) is an antimetabolite chemotherapy used for a variety of solid tumors. It has the potential to cause a wide spectrum of cardiotoxicity, ranging from asymptomatic electrocardiographic changes to cardiomyopathy and subsequent cardiac failure. Main body of the abstract: We present two descriptive cases of new-onset severe cardiomyopathy induced by 5-FU followed by a review of the literature.

**Conclusion:**

Our case series emphasizes the importance of early recognition of this rare complication and prompt cessation of 5-FU, as cardiac dysfunction in this context is potentially reversible.

## Background

5-fluorouracil (5-FU) is a fluoropyrimidine (FP) antimetabolite agent used in a variety of solid tumors treatment. A potential severe side effect of 5-FU is cardiotoxicity, which often presents with chest pain related to coronary vasospasm. More serious cardiotoxicity, including dilated cardiomyopathy, ventricular arrhythmia, and sudden cardiac death has also been reported in the literature [1–4]. 5-FU cardiotoxicity is only second to anthracyclines in terms of incidence of cardiotoxicity [5, 6]. Further, cardiotoxicity induced by 5-FU carries a high risk of morbidity and mortality if left unrecognized [7].

## Main text

### Case presentations

#### Case 1

**Fig. 1:**
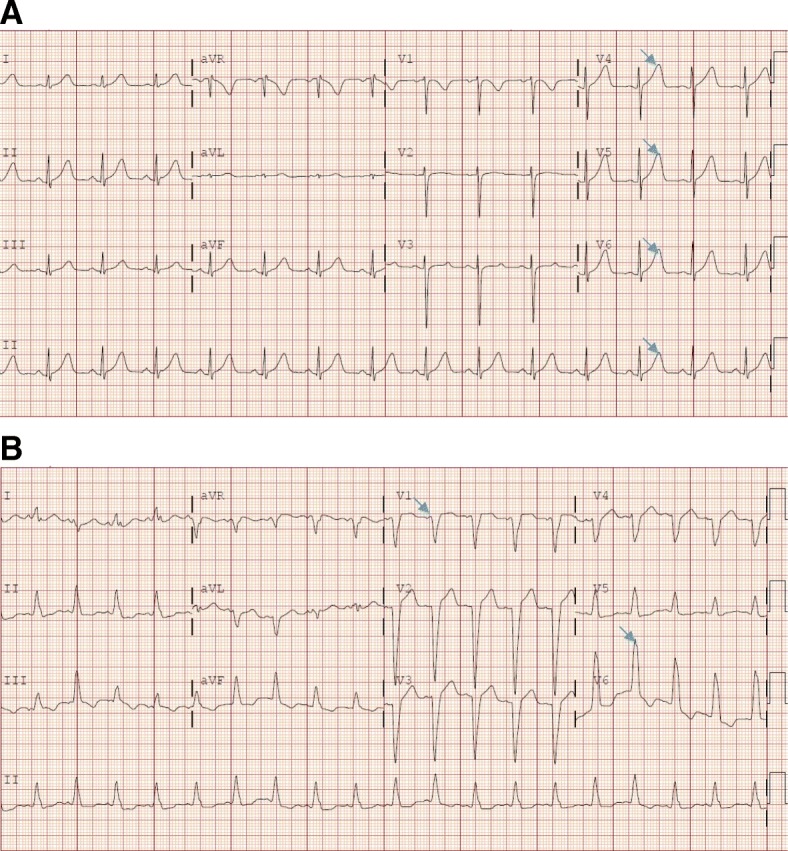
**a** 12 lead ECG from Case 1 showing hyperacute T waves (arrows) with no ST elevation or depression. **b**: 12 lead ECG from case 2 showing sinus tachycardia and a new left bundle branch block (arrows)

A 47-year-old woman with no known history, or risk factors, of cardiac disease, was diagnosed with stage III colon adenocarcinoma. After undergoing laparoscopic ileocolectomy, the patient was started on adjuvant chemotherapy with modified FOLFOX6 (fluorouracil, leucovorin, and oxaliplatin). 5-FU was given as bolus at 400 mg/m2, followed by 1200 mg/m2/day continuous infusion over 46 h. Approximately 12 h into receiving the first infusional dose of 5-FU, the patient developed progressive substernal chest pain and shortness of breath. Electrocardiography (ECG) revealed hyperacute T waves with no ST elevation or depression (Fig. 1a). Initially, cardiac biomarkers indicated a mildly elevated troponin I at the level of 0.05 ng/ml (normal range < 0.04 ng/ml), with a peak level of 0.14 ng/ml at 48 h. Echocardiogram on the following day revealed severely reduced left ventricular function with an ejection fraction (EF) of 20–25% with severe hypokinesis of the entire left wall. One month earlier the patient had an unremarkable echocardiogram. Subsequent coronary CT revealed normal coronaries with no stenosis. A diagnosis of 5-FU induced cardiomyopathy was made. 5-FU was subsequently discontinued. The patient was seen by a cardio-oncologist and was placed on a beta blocker and ACE inhibitor. Repeat echocardiogram six weeks later revealed normalization of left ventricular function with an EF of 55–60%. The patient was subsequently given one cycle of capecitabine, which she tolerated. However, she was then admitted to hospital multiple times due to other reasons. A decision was made no further chemo should be given since the patient was 12 weeks from surgery.

#### Case 2

A 58-year-old woman was diagnosed with stage IV colon adenocarcinoma with metastases. The patient had no history, or risk factors, of cardiac disease. After palliative laparoscopic end loop colostomy, the patient was started on palliative chemotherapy with modified FOLFOX6. 5-FU was given as bolus at 400 mg/m2, followed by 1200 mg/m2/day continuous infusion over 46 h. After 3 cycles of treatments, she presented to the emergency department with severe dyspnea and cough. ECG revealed tachycardia and a new left bundle branch block (Fig. 1b). The cardiac biomarker troponin I was negative. Echocardiogram showed severe decreased left ventricular with an ejection fraction of 20–25% with severe global hypokinesis. No previous echocardiogram was available for comparison. It was determined based on the acute onset of symptoms and the lack of previous cardiac symptoms that the patient developed 5-FU induced cardiomyopathy. 5-FU was subsequently discontinued. The patient was evaluated by a cardio-oncologist and placed on a beta blocker and ACE inhibitor. A repeat echocardiogram seven months later, unfortunately, showed persistent severe left ventricular dysfunction with an EF 15–20%. Continued treatment for the cancer included IROX (irinotecan and oxaliplatin) to avoid further 5-FU exposure.

## Discussion

5-FU is a fluoropyrimidine antimetabolite agent used broadly in the treatment of a variety of solid tumors. The more common side effects of 5-FU include diarrhea, mucositis, and myelosuppression. Cardiotoxicity is a more serious side effect ranging from asymptomatic ECG changes to life-threatening cardiogenic shock [3, 4, 8].

### Epidemiology and risk factors (Table 1)

The reported incidence of cardiotoxicity ranges from 1 to 18% of patients exposed to fluoropyrimidine [9–15]. Such wide variation could be a reflection of different risk profiles in the study population and also the different administration schedules. The risk of cardiotoxicity is reported to be increased in patients with concurrent chest wall radiation therapy [16], multi-agent chemotherapy [12], and pre-existing cardiac disease (i.e., coronary artery disease [CAD], structural heart disease, and cardiomyopathy) [9, 10, 17]. However, known risk factors for ischemic heart disease such as smoking, diabetes mellitus, obesity, hypertension, and hyperlipidemia do not appear to be associated with the development of cardiotoxicity [1, 11, 12, 18].

**Table 1:** Incidence of Cardiotoxicity According to Regimen of Fluoropyrimidine

Author	Cancer Studied	5-FU regimen used	Number of patients	Overall 5-FU induced cardiotoxicity incidence (*N*)	Signs and symptoms
Polk et al.	Breast cancer	Capecitabine^a^	452	4.9% (22)	Chest pain, dyspnea
Jensen et al.	Colorectal cancer	FOLFOX4^b^	106	8.5% (9)	Angina
Holubec et al.	Colorectal cancer	de Gramont regimen^c^ FOLFIRI^d^	42	57% (24)	Elevated cardiac biomarkers
Yilmaz et al.	GI cancer	de Gramont^c^	27	7.4% (2)	Angina
Turan et al.	Not specified	Not specified	32	12.5% (4)	Angina, ECG changes
Ng et al.	Colorectal cancer	XELOX^e^	153	6.5% (10)	Angina, Heart failure, Sudden cardiac death
Meydan et al.	GI, Breast, and Head and Neck cancers	de Gramont regimen^c^	231	3.9% (9)	Acute coronary syndrome, heart failure, cardiac arrhythmia

^a^Capecitabine: 1000 mg/m^2^ orally twice daily

^b^FOLFOX4: oxaliplatin 85 mg/m^2^ IV, leucovorin 200 mg/m^2^ IV, 5-FU IV bolus 400 mg/m^2^ followed by continuous IV infusion 5-FU 600 mg/m^2^ over 22 h

^c^de Gramont regimen: leucovorin 200 mg/m^2^ IV, 5-FU bolus 400 mg/m^2^ and 5-FU 600 mg/m^2^ continuous IV infusion over 22 h

^d^FOLFIRI: irinotecan 180 mg/m^2^ IV, leucovorin 400 mg/m^2^ IV, 5-FU IV bolus 400 mg/m^2^ followed by 5-FU 2400 mg/m^2^ continuous IV infusion over 46 h

^e^XELOX: capecitabine 1000 mg/m^2^ two times per day on day 1–14, oxaliplatin 130 mg/m^2^ IV on day 1

Protracted infusion of 5-FU is a well-recognized risk factor for patients to develop cardiotoxicity [8, 11, 19–21]. A review of 377 cases of 5-FU related cardiotoxicity confirmed that the majority of cases of cardiotoxicity occur in the setting of continuous infusion [3]. In a retrospective study comparing different chemotherapy regimens with 5-FU used to treat colorectal and gastric cancer, patients receiving continuous infusion 5-FU therapy had a reported incidence of cardiotoxicity as high as 10–18%. This is in contrast to a 5% rate of cardiotoxicity in patients receiving 5-FU in a bolus fashion [8]. In another study evaluating 1000 patients receiving 5-FU, the rates of cardiotoxicity were similarly lower (1.6–3%) with bolus administration of 5-FU [9]. The likely reason for differences in cardiotoxicity with continuous versus bolus infusion is that the half-life of 5-FU is 15–20 min and thus the drug is rapidly cleared when given in bolus fashion [22].

Alternatively, capecitabine, an orally available 5-FU prodrug, was found to have an incidence of cardiotoxicity of 3–9%, which is similar to that of continuous 5-FU infusion therapy [11, 18, 22]. TAS-102 (Lonsurf) is an oral cytotoxic drug that has a nucleoside analog (trifluridine) and a thymidine phosphorylase inhibitor (tipiracil) approved for use in refractory metastatic colorectal cancer. In the registration phase III study that led to its FDA approval, only one patient treated with TAS-102 was reported to have an episode of cardiac ischemia (unknown mechanism and attribution) among 800 treated patients [23]. In a recent review, Petrelli et al. suggest that TAS-102 could represent an alternative option for patients with increased risk factors of developing cardiac events [24]. S-1 is a drug that contains fluorouracil prodrug tefagur and gimeracil used in gastric cancer. In the published phase II or III studies of S-1, no grade III or IV cardiovascular events were reported [25–27]. S-1 currently is not available in the USA.

It is also important to point out that asymptomatic ECG changes have been reported to be as high as 88% [17]. 5-FU chemotherapy is commonly administered in the outpatient setting, and patients do not require routine cardiac monitoring. Thus, subclinical ECG changes and asymptomatic myocardial injury are likely an underreported phenomenon.

### Clinical manifestations (Table 2)

Angina is the most common manifestations of fluoropyrimidine-induced cardiotoxicity, which can occur in up to 19–45% of patients with or without ST and T wave ECG changes [3, 28]. Wacker et al. reported in a case series that up to 19% of the patients experienced angina during treatment, with episodes lasting up to 12 h after cessation of drug infusion [28].

**Table 2:** Potential Clinical and ECG Manifestations of Fluoropyrimidine Induced Cardiotoxicity

Clinical	ECG
Myocardial infarction	Supraventricular tachycardia
Cardiomyopathy	Ventricular tachycardia
Myocarditis	QT prolongation
Pericarditis	Ischemic changes (i.e., ST and T wave abnormalities)
Coronary dissection	
Sudden cardiac death	

Less commonly, myocardial infarction, congestive heart failure, and reversible cardiomyopathy have been reported [1, 29–31]. In one report, the incidence of cardiomyopathy with left ventricular dysfunction was estimated to be 2% [3]. Severe cardiotoxic manifestations have been reported in case reports, including coronary dissection, ventricular tachyarrhythmia, cardiogenic shock (requiring intra-aortic balloon pump and extracorporeal membrane oxygenation support), and sudden cardiac death [32–34].

Aside from symptomatic cardiotoxicity, some patients may develop silent cardiac ischemia. In a prospective study, Rezkalla et al. identified an association between ischemic ECG changes and 5-FU infusion in otherwise asymptomatic patients who underwent ambulatory rhythm monitoring [17]. Other arrhythmias including QT prolongations, and less commonly torsades de pointes have been reported [17, 28, 35].

### Mechanisms of cardiotoxicity (Table 3)

The precise mechanism of cardiotoxicity from fluoropyrimidines remains unclear, but several mechanisms have been proposed, including coronary artery vasospasm, direct toxicity to the myocardium, endothelial dysfunction, and a hypercoagulable state causing thrombosis. It should be noted that these mechanisms were elucidated through animal modeling, case reports, and small clinical studies.

**Table 3:** Proposed Mechanisms of Fluoropyrimidine Induced Cardiotoxicity

Coronary Vasospasm	Direct Myocardial Injury	Vascular Endothelial Dysfunction	Impaired Oxygen Delivery
● Protein kinase C ● Endothelin-I	● Alpha-fluoro-beta-alanine (FBAL) (breakdown product of 5-FU)	● Microthrombotic occlusions resulting from direct toxic effect of 5-FU on vascular endothelial cells ● Oxygen free radicals	● Erythrocyte membranes change leading to diminished ability to deliver oxygen

### Coronary vasospasm

Coronary vasospasm leading to an acute ischemic event is probably the most well recognized cardiac side effect of fluoropyrimidines. Patients present with signs and symptoms of acute coronary syndrome and ECG often reveals ST segment changes along with a rise in cardiac biomarkers, such as troponin. However, coronary angiography is typically normal without evidence of a thrombotic event [36]. Mosseri et al. found that protein kinase C may be a mediator of 5-FU-induced vasoconstriction, and demonstrated endothelium-independent vasoconstriction of rabbit aortic rings with increasing doses of 5-FU [37]. Additionally, Thyss et al. described high plasma levels of endothelin-1 in patients that experienced 5-FU-induced cardiotoxicity [38]. Endothelin-1 is a potent vasoconstrictor that has a known regulatory role in vascular vasomotor tone in coronary artery disease [39, 40]. The efficacy of vasodilator therapy including non-dihydropyridine calcium channel blockers such as verapamil and nitrates were noted to be effective in resolving chest pain and dynamic ECG changes in the setting of 5-FU induced cardiotoxicity [41, 42]. These data support that theory of coronary vasoconstriction as part of the pathophysiology of fluoropyrimidine-induced cardiotoxicity. As noted earlier in this manuscript coronary vasospasm is seen with continuous infusion of 5-FU, or alternatively with the fluoropyrimidine, capecitabine.

### Direct myocardial injury

Another possible mechanism of cardiotoxicity is a direct toxic effect of fluoropyrimidine on cardiomyocytes, as evidenced by global systolic dysfunction, which does not correspond to any individual coronary artery territory [10]. An animal study of rabbits demonstrated 5-FU induced diffuse myocarditis with necrosis [43]. Sarcoplasmic reticulum dilatation was demonstrated on one case report utilizing ventricular biopsy, which is similar to the mechanism of doxorubicin-associated cardiomyopathy [44]. One of the down-stream degradation compounds of 5-FU is alpha-fluoro-beta-alanine (FBAL), which appears to serve as an important mediator of the direct toxic effect [45, 46]. In a case report, Muneoka et al. demonstrated elevated levels of FBAL in a patient after 5-FU induced myocardial infarction. The patient was then treated with the prodrug S-1, an oral fluoropyrimidine that does not metabolize to FBAL, without recurrent cardiac adverse effects [46].

### Vascular Endothelial Dysfunction & Impaired Oxygen Delivery

Vascular dysfunction with microthrombi formation has been shown to be a potential mechanism that results in fluoropyrimidine-induced cardiotoxicity. The microthrombotic occlusion is usually not detectable by coronary angiography [45]. Several animal studies examined the direct toxic effects of 5-FU on vascular endothelial cells and noted direct endothelial damage and subsequent platelet and fibrin accumulation, which was confirmed by electron microscopy [47, 48]. Experimental studies also suggest that the use of anticoagulation therapy may partially mitigate this toxicity [2, 49]. Some authors, on the other hand, believe free oxygen radicals also play a role in cytotoxic endothelial dysfunction [50].

Spasojevic et al. demonstrated that 5-FU causes changes to the erythrocyte membranes leading to increased fluidity and conversion of the erythrocyte from its usual biconcave shape to an echinocyte shape [51, 52]. The resulted membrane changes diminish the erythrocyte’s ability to deliver and transport oxygen, resulting in myocardial ischemia and injury.

### Treatment

There is no standard treatment recommendation for fluoropyrimidine induced cardiotoxicity. The current consensus is that 5-FU should be discontinued as soon as potential cardiotoxicity is suspected. Upon fluoropyrimidine discontinuation, the patient should be treated symptomatically with antianginal therapy such as nitrates and non-dihydropyridine calcium channel blocker as empiric treatment for acute coronary vasospasm. It is reported that this approach has been shown to abort symptoms in up to approximately 70% of patients [3, 8].

Multidisciplinary discussion between oncology and cardiology is critical in managing cancer patients with suspected 5-FU associated cardiotoxicity. Ensuring that symptoms have completely resolved before attempting further chemotherapy. These patients should be managed with risk stratification and treatment per ACC/AHA guidelines. As preexisting CAD is a known risk factor for cardiotoxicity, risk reduction strategies with smoking secession, optimizing blood pressure, statin use and aggressive diabetes control should be implemented [45].

In 2015, uridine triacetate was approved by the Food and Drug Administration (FDA) as an antidote to 5-FU (or capecitabine) overdose or for those who exhibit early-onset, severe or life-threatening toxicity affecting the cardiac or central nervous system, and/or early onset, unusually severe adverse reactions (e.g. gastrointestinal toxicity and/or neutropenia) [53]. Uridine triacetate is an oral active prodrug of uridine, which is a naturally occurring nucleotide and competes with the 5-FU metabolite, for incorporation into RNA of normal tissue. It was shown in a small study that it has superior survival rate in treated patients who experienced life-threatening toxicity from 5-FU when compared to supportive care only in a historical case cohort [54]. However, due to the limitation of the study, further studies are needed to clarify its role of in the treatment of fluoropyrimidine associated cardiotoxicity.

In some reports, the cardiotoxicity appears to be reversible after cessation of therapy as demonstrated by our first case. This is especially true in patients without underlying cardiac disease and when vasospasm alone induces a hibernating myocardium that recovers with adequate time and reperfusion [5]. Thus, high clinical awareness of this problem is critical, as early cessation of therapy may prevent further damage and allow for potential reversal of damage. Referral to a cardiac specialist, ideally a cardio-oncologist, is prudent for medical optimization and vigilant monitoring.

### Prophylaxis and prevention

Several pharmacological interventions have been assessed as preventive strategies with mixed results. A study done by Eskilsson et al. pursued prophylactic administration of verapamil, a non-dihydropyridine calcium channel blocker, to prevent 5-FU induced vasospasm. The study did not show a benefit for verapamil in this capacity [55]. On the other hand, Ambrosy et al. reported that five patients who previously experienced chest pain and dyspnea with their initial doses of capecitabine had resolution of any further symptoms with co-administration of diltiazem [56]. Nifedipine was also found in a case report as an effective agent to prevent 5-FU induced coronary vasospasm when the patient was receiving continuous 5-FU infusion for gastric cancer [57]. Nitrates have also been tried with variable success [58, 59]. There is currently no randomized trial evaluating the role of calcium channel blocker or nitrates in this setting. These agents could be used in selected situations on a case by case basis.

Patients re-challenged with 5-FU therapy after the initial insult have a risk of recurrence of cardiotoxicity reported to be as high as 82 to 100% [60], and death can be up to 13% [3, 10]. Important considerations before restarting chemotherapy are if the agent in question provides the best chances for survival with cancer treatment and if there is an alternative chemo regimen that can be used. As mentioned above, TAS-102 may be an alternative option for patients with increased risk factors of developing cardiac events with colorectal cancer [24].

Fluoropyrimidine reintroduction can be considered if it is deemed the most efficacious choice per multidisciplinary discussion and patients are well informed about the benefits and risks of resuming therapy. There is limited evidence supporting switching to 5-FU bolus in patients who experienced cardiotoxicity with infusional 5-FU or capecitabine [58, 61, 62]. Jensen et al. reported significantly decreased cardiotoxicity in 9 out of 12 patients receiving prophylaxis with either a beta-blocker, calcium channel blocker, or long-acting nitrate as well as dose-reduced fluoropyrimidine [8]. Outpatient ECGs monitoring may be important to implement in patients with reintroduced therapy to detect silent ischemia and arrhythmia [17, 63].

## Conclusion

Cardiotoxicity induced by fluoropyrimidines is a clinically relevant side effect that all oncologists should be familiar with, as early recognition is crucial to prevent significant morbidity. The mechanisms for cardiotoxicity include coronary vasospasm, direct myocardial injury, vascular endothelial dysfunction, and impaired oxygen delivery. There is currently no consensus on the optimal treatment and/or prophylaxis of these complications, aside from early detection and cessation of the offending agent. Vigilant monitoring for cardiotoxicity with early cessation and supportive care intervention remains the most effective treatment with some opportunity for cardiac recovery.

## Data Availability

Not applicable

## Abbreviations

5-FU: 5-Fluorouracil
ACC: American college of cardiology
ACE inhibitors: Angiotensin-converting enzyme inhibitors
AHA: American heart association
CAD: Coronary heart disease
CT: Computerized tomography
ECG: Electrocardiography
EF: Ejection fraction
FBAL: Alpha-fluoro-beta-alanine
FDA: Food and Drug Administration
FOLFOX6: fluorouracil, leucovorin, and oxaliplatin
FP: fluoropyrimidine
IROX: Irinotecan and oxaliplatin
TAS 102: Trifluridine and tipiracil
